# Natural history and outcomes in paediatric RASopathy‐associated hypertrophic cardiomyopathy

**DOI:** 10.1002/ehf2.14637

**Published:** 2024-01-13

**Authors:** Olga Boleti, Gabrielle Norrish, Ella Field, Kathleen Dady, Kim Summers, Gauri Nepali, Vinay Bhole, Orhan Uzun, Amos Wong, Piers E. F. Daubeney, Graham Stuart, Precylia Fernandes, Karen McLeod, Maria Ilina, Muhammad Najih Liaqath Ali, Tara Bharucha, Grazia Delle Donne, Elspeth Brown, Katie Linter, Caroline B. Jones, Jonathan Searle, William Regan, Sujeev Mathur, Nicola Boyd, Zdenka Reinhardt, Sophie Duignan, Terence Prendiville, Satish Adwani, Juan Pablo Kaski

**Affiliations:** ^1^ Centre for Inherited Cardiovascular Diseases, Department of Cardiology Great Ormond Street Hospital London UK; ^2^ Institute of Cardiovascular Science University College London London UK; ^3^ The Heart Unit Birmingham Children's Hospital Birmingham UK; ^4^ Children's Heart Unit University Hospital of Wales Cardiff UK; ^5^ Department of Paediatric Cardiology Royal Brompton and Harefield NHS Trust London UK; ^6^ Department of Paediatric Cardiology Bristol Royal Hospital for Children Bristol UK; ^7^ Department of Paediatric Cardiology Royal Hospital for Children Glasgow UK; ^8^ Department of Paediatric Cardiology Southampton General Hospital Southampton UK; ^9^ Department of Paediatric Cardiology Leeds General Infirmary Leeds UK; ^10^ Department of Paediatric Cardiology Glenfield Hospital Leicester UK; ^11^ Department of Cardiology Alder Hey Children's Hospital Liverpool UK; ^12^ Children's Heart Service Evelina Children's Hospital London UK; ^13^ Department of Paediatric Cardiology The Freeman Hospital Newcastle UK; ^14^ The Children's Heart Centre Our Lady's Children's Hospital Dublin Ireland; ^15^ Department of Paediatric Cardiology John Radcliffe Hospital Oxford UK

**Keywords:** Hypertrophic cardiomyopathy, Paediatric cardiology, Inherited cardiac conditions, Genetics, RASopathy, Noonan, Predictors, Arrhythmia, Sudden cardiac death, Mortality

## Abstract

**Aims:**

This study aimed to describe the natural history and predictors of all‐cause mortality and sudden cardiac death (SCD)/equivalent events in children with a RASopathy syndrome and hypertrophic cardiomyopathy (HCM).

**Methods and results:**

This is a retrospective cohort study from 14 paediatric cardiology centres in the United Kingdom and Ireland. We included children <18 years with HCM and a clinical and/or genetic diagnosis of a RASopathy syndrome [Noonan syndrome (NS), NS with multiple lentigines (NSML), Costello syndrome (CS), cardiofaciocutaneous syndrome (CFCS), and NS with loose anagen hair (NS‐LAH)]. One hundred forty‐nine patients were recruited [111 (74.5%) NS, 12 (8.05%) NSML, 6 (4.03%) CS, 6 (4.03%) CFCS, 11 (7.4%) Noonan‐like syndrome, and 3 (2%) NS‐LAH]. NSML patients had higher left ventricular outflow tract (LVOT) gradient values [60 (36–80) mmHg, *P* = 0.004]. Over a median follow‐up of 197.5 [inter‐quartile range (IQR) 93.58–370] months, 23 patients (15.43%) died at a median age of 24.1 (IQR 5.6–175.9) months. Survival was 96.45% [95% confidence interval (CI) 91.69–98.51], 90.42% (95% CI 84.04–94.33), and 84.12% (95% CI 75.42–89.94) at 1, 5, and 10 years, respectively, but this varied by RASopathy syndrome. RASopathy syndrome, symptoms at baseline, congestive cardiac failure (CCF), non‐sustained ventricular tachycardia (NSVT), and maximal left ventricular wall thickness were identified as predictors of all‐cause mortality on univariate analysis, and CCF, NSVT, and LVOT gradient were predictors for SCD or equivalent event.

**Conclusions:**

These findings highlight a distinct category of patients with Noonan‐like syndrome with a milder HCM phenotype but significantly worse survival and identify potential predictors of adverse outcome in patients with RASopathy‐related HCM.

## Introduction

Hypertrophic cardiomyopathy (HCM) is a disease of the heart muscle characterized by unexplained left ventricular (LV) hypertrophy (LVH).^1^ Most cases of HCM are caused by variants in the cardiac sarcomere protein genes,^2^ but the aetiology of childhood HCM is heterogeneous and ~30% of cases are related to inborn errors of metabolism, neuromuscular disorders, and malformation syndromes.^3^ In particular, RASopathy syndromes account for nearly 20% of childhood‐onset HCM cases.^4^, ^5^

The RASopathies are a group of developmental disorders caused by germline variants in genes encoding proteins involved in the RAS–mitogen‐activated protein kinase (MAPK) pathway and are among the commonest malformation syndromes, with a prevalence of ~1 in 1000–2500 children.^6^, ^7^, ^8^ They include Noonan syndrome (NS), NS with multiple lentigines (NSML, previously known as LEOPARD syndrome), Costello syndrome (CS), cardiofaciocutaneous syndrome (CFCS), and NS with loose anagen hair (NS‐LAH).^7^, ^9^, ^10^, ^11^, ^12^ These conditions are characterized by facial dysmorphia, failure to thrive, short stature, skeletal malformations, variable cognitive defects, and an increased risk of solid tumours.^13^ Cardiac defects are reported in over 80% of cases, most commonly pulmonary valve stenosis and HCM.^14^, ^15^, ^16^, ^17^, ^18^ Although, histologically, RASopathy‐related HCM is indistinguishable from HCM caused by sarcomere protein gene variants, with myocyte disarray and fibrosis,^19^, ^20^ the clinical presentation and natural history can be substantially different.^4^, ^21^ Despite these differences, the clinical management and risk stratification of patients with RASopathy‐related HCM are currently extrapolated from that of sarcomeric HCM, and specific clinical evaluation and management guidelines for RASopathy‐associated HCM have not been developed. An improved understanding of the relationship between aetiology, phenotype, and outcomes is necessary in order to optimize clinical care in this distinct population.

The aim of this study was to describe the clinical features, outcomes, and predictors of all‐cause mortality and sudden cardiac death (SCD) or equivalent events in a large, multicentre national cohort of patients with RASopathy‐associated HCM diagnosed ≤18 years.

## Methods

### Study population

The study cohort consisted of patients ≤18 years with HCM and a clinical and/or genetic diagnosis of a RASopathy syndrome (NS, NSML, CS, CFCS, and NS‐LAH), consecutively evaluated between 1 January 1985 and 31 December 2020, in all 14 paediatric cardiology centres in the United Kingdom (see Supporting Information, *Table* *S1*). Patients with clinical features of a RASopathy syndrome not fulfilling diagnostic criteria for one of the previously described syndromes and without a pathogenic (P)/likely pathogenic (LP) variant were labelled ‘Noonan‐like syndrome’. A diagnosis of HCM was defined as an LV wall thickness >2 standard deviations above the body surface area‐corrected population mean (*z* score ≥2) that could not be explained solely by abnormal loading conditions.^1^ The authors from each participating centre guaranteed the integrity of data from their institution. Eligible patients were identified by the principal investigator at each collaborating site. Data were collected independently at each participating centre, and each local investigator provided data on all consecutive patients with RASopathy‐associated HCM from their centre.

### Patient assessment and data collection

Anonymized, non‐invasive clinical data were collected retrospectively, including demographics, family history of HCM/SCD, extra‐cardiac manifestations of each RASopathy syndrome, syndrome, genetic analysis results, heart failure symptoms [New York Heart Association (NYHA)/Ross functional classification^22^, ^23^], medication, resting and ambulatory 12‐lead electrocardiogram, and two‐dimensional Doppler and colour transthoracic echocardiogram (from contemporaneously written reports). Age at diagnosis was defined as the age at which HCM was first diagnosed, which may have been prior to the patient(s) being seen for the first time in a paediatric cardiology service. Data were collected from the first assessment and the last clinical follow‐up in a paediatric cardiology centre. End of follow‐up was defined as last clinical follow‐up or transition to adult services, whichever came first; data following transition to adult services were not available for analysis.

Electrocardiographic criteria for LVH were based on the Sokolow–Lyon criteria.^24^ Previously published normal values for age were employed for QRS axis and electrocardiographic intervals.^25^ The following findings were considered abnormal on the electrocardiogram (ECG): axis deviation, evidence of right atrial (RA) or left atrial (LA) dilatation, criteria for right ventricular hypertrophy (RVH)/LVH, left bundle branch block (LBBB)/right bundle branch block (RBBB) morphology, and T‐wave inversion outside of the normal variants for age (classed by location: inferior, lateral, and anterior). Echocardiographic analysis was performed in line with the American Society of Echocardiography guidelines,^26^ and measurements were taken according to current guidelines.^1^ Maximal LV wall thickness (MLVWT) was defined as the maximal myocardial thickness as measured by echocardiography in any of the LV segments.^1^ LV outflow tract (LVOT) obstruction (LVOTO) was defined as a peak instantaneous gradient ≥30 mmHg.^1^ Right ventricular outflow tract (RVOT) obstruction (RVOTO) was defined as a peak instantaneous gradient ≥36 mmHg.^27^ Impaired LV systolic function was defined as a fractional shortening (FS) ≤28% or ejection fraction ≤55%^27^ and impaired diastolic function as an average E/E′ ratio of >14.^28^

### Clinical outcomes

The primary clinical outcome was all‐cause mortality [congestive cardiac failure (CCF), SCD, other cardiovascular (CVS) death, and non‐CVS death]. Secondary outcomes included SCD or equivalent event [appropriate implantable cardioverter defibrillator (ICD) therapy, aborted cardiac arrest, or sustained ventricular tachycardia (VT) with haemodynamic compromise]. Data on atrial arrhythmias, CCF admissions to hospital, ICD implantation, cardiac transplantation, and surgical/catheter‐based interventions at follow‐up were also collected. Outcomes were determined by the treating cardiologist at each site.

### Genetics

Patients were diagnosed with a RASopathy syndrome clinically and/or after genetic testing. Genetic testing was performed at the treating clinician's discretion. In patients in whom genetic testing had been performed, the following data were collected: date of testing, size of gene panel, and variants identified (gene and protein change).

### Statistical analysis

Body surface area was calculated from weight.^29^ MLVWT and LA diameter measurements are expressed in millimetres and as body surface area‐corrected *z* scores.^30^, ^31^ Continuous variables are described as mean (±standard deviation) or median [inter‐quartile range (IQR)], with three group comparisons conducted using analysis of variance (ANOVA) or Kruskal–Wallis tests, respectively. The distribution of categorical variables was compared using the *χ* ^2^ test or Fisher's exact test. A significance level of 0.05 was used for all comparisons. The follow‐up time for all patients was calculated from the date of their first evaluation to the date of reaching the study endpoint, death from another cause, or the date of their most recent evaluation prior to the end of the study period. Age at first assessment was categorized for analysis purposes: <6 months, 6–12 months, 12 months to 5 years, and >5 years. Era of presentation was categorized for analysis purposes: 1985–99, 2000–10, and 2010–20. Percentages expressed are based on available values.

Estimates of survival were obtained using the Kaplan–Meier product limit method. The association of clinical variables with the outcome of interest was assessed in a univariate Cox proportional hazard model. Mortality and cardiac transplantation were censoring events for survival analyses in this study. All statistical analyses were performed with STATA (Stata Statistical Software Release 17; StataCorp LP, College Station, TX).

### Ethics

This study complies with the Declaration of Helsinki. Local ethical approval was obtained at each participating site with waiver of informed consent for retrospective, anonymized data. The data underlying this article cannot be shared publicly as consent for dissemination of patient data was not obtained.

## Results

### Demographics and presentation

One hundred forty‐nine patients with a RASopathy syndrome and HCM were identified, of whom 92 (61.7%) were male. One hundred eleven (74.5%) were diagnosed with NS, 12 (8.1%) with NSML, 6 (4%) with CS, 6 (4%) with CFCS, 11 (7.4%) with Noonan‐like syndrome, and 3 (2%) with NS‐LAH. Sixty‐nine patients (65.1%) had one or more extra‐cardiac manifestations (see Supporting Information, *Figure* *S1*). Seventeen (11.5%) had a family history of HCM. Sixty‐seven patients (60.9%) had concomitant congenital heart defects (CHDs), of whom 32 (29.1%) had more than one CHD (see Supporting Information, *Table* *S2*). The median age of diagnosis of HCM was 1.38 (IQR 0–10.28) months, while the median age at first assessment was 22.46 (IQR 5.67–82.89) months. The age category according to RASopathy syndrome is shown in Supporting Information, *Figure* *S2*. Demographics and baseline clinical characteristics are summarized in *Table* *1*. Clinical characteristics of the 11 patients with Noonan‐like syndrome are individually shown in Supporting Information, *Table* *S3* and, for the three patients with NS‐LAH, are detailed separately in Supporting Information, *Table* *S4*. Patients with variants in *PTPN11* and *RIT1* had a higher proportion of CHD and an earlier age at diagnosis (Supporting Information, *Table* *S5*). There were no significant differences in clinical parameters across different eras (Supporting Information, *Table* *S6*).

**Table 1:** Demographics and baseline characteristics

	Total	NS	NSML	CS	CFCS	Noonan‐like	*P* value
Number of patients, *n* (%)	149 (100)	111 (74.5)	12 (8.1)	6 (4)	6 (4)	11 (7.4)	—
Gender (male), *n* (%)	92 (61.7)	70 (60.1)	9 (75)	3 (50)	1 (16.7)	6 (54.5)	0.163
Age at diagnosis (months), median (25–75th centile)	1.4 (0–10.3)	1.28 (0–8.7)	0 (0–11)	3.3 (2.4–71.2)	−0.16 (−0.3 to 6.7)	4.9 (−1.2 to 121.9)	0.401
Age at baseline (months), median (25–75th centile)	22.5 (5.7–82.9)	26.4 (6.4–83.7)	37.7 (3–129.6)	13.6 (9.6–27.1)	8.11 (0.9–15.4)	14.1 (1.2–64)	0.563
Proband, *n* (%)	121 (90.3)	91 (82)	9 (75)	6 (100)	5 (83.3)	10 (90.1)	0.269
FHx HCM, *n* (%)	17 (11.4)	12.6 (14)	3 (25)	6 (100)	—	—	0.223
PMHx CCF, *n* (%)	23 (22.2)	16 (14.4)	5 (41.7)	—	—	2 (18.2)	0.104
PMHx arrhythmia, *n* (%)	7 (7.1)	6 (5.4)	—	—	—	1 (9.1)	0.729
CHD, *n* (%)	51 (46.4)	38 (34.2)	4 (33.3)	—	3 (50)	4 (36.4)	0.174
Extra‐cardiac manifestations	69 (65.1)	54 (48.6)	5 (41.7)	3 (50)	3 (50)	4 (36.4)	**0.001**
Symptoms, *n* (%)	61 (57.3)	50 (45.1)	7 (58.3)	1 (16.7)	1 (16.7)	2 (18.2)	**0.073**
Shortness of breath	67 (82.8)	58 (81.69)	6 (85.71)	—	1 (16.67)	2 (66.67)	**0.071**
Fatigue	10 (18.03)	8 (11.26)	1 (14.28)	—	—	1 (33.33)	0.121
Presyncope/syncope	3 (4.91)	3 (4.22)	—	—	—	—	0.354
Chest pain/palpitations	2 (3.28)	2 (2.81)	—	—	—	—	0.784
Medications, *n* (%)	69 (47.9)	50 (45.1)	9 (75)	1 (16.7)	3 (50)	5 (45.5)	0.198
Beta‐blockers	56 (81.15)	42 (84)	8 (88.89)	1 (100)	1 (33.33)	4 (66.67)	0.134
Diuretics	12 (17.39)	9 (18)	—	—	2 (66.67)	1 (16.67)	0.151
Disopyramide	4 (5.79)	3 (6)	1 (11.11)	—	—	—	0.702
Ca channel blockers	3 (4.34)	1 (2)	1 (11.11)	—	—	1 (16.67)	0.264
Amiodarone	1 (1.44)	1 (2)	—	—	—	—	0.987

CCF, congestive cardiac failure; CFCS, cardiofaciocutaneous syndrome; CHD, congenital heart defect; CS, Costello syndrome; FHx, family history; HCM, hypertrophic cardiomyopathy; NS, Noonan syndrome; NSML, Noonan syndrome with multiple lentigines; PMHx, past medical history.Bold numbers represent statistically‐significant p values

### Genetics

Genetic testing was performed in 117 patients (78.5%), with a P or LP variant identified in 81 (69.2%). The most commonly implicated gene was *PTPN11* (*N* = 28, 34.6%), followed by *RAF1* (*N* = 18, 22.2%), *RIT1* (*N* = 8, 9.9%), and *HRAS* (*N* = 8, 9.9%). Five patients (4.3%) had an additional variant identified [*RAF1* (P) and *MYH7* (VUS); *PTPN11* (P) and *MYH7* (VUS); *PTPN11* (P) and *MYH7* (LP); *KRAS* (LP) and *MEK1* (VUS); and *LZTR1* (LP) and *HRAS* (VUS)]. Supporting Information, *Figure* *S3* shows the frequency of implicated genes according to RASopathy syndrome. Specific nucleotide and protein changes are detailed in Supporting Information, *Table* *S7*. The proportion of patients undergoing genetic testing, and the subsequent yield of genetic testing, increased over time (Supporting Information, *Table* *S6*).

### Echocardiographic characteristics

Data from the echocardiogram at first assessment in a paediatric cardiology centre were available in 116 patients (77.9%). Forty‐six patients (48.9%) had biventricular hypertrophy, 44 (45.8%) had LVOTO, and 18 (39.1%) had RVOTO. Nine patients (30%) had evidence of diastolic impairment at first assessment. Echocardiographic data are summarized in *Table* *2*, and a comparison of the echocardiographic phenotype among the most prevalent genes is presented in Supporting Information, *Table* *S8*.

**Table 2:** Echocardiographic features

	Total	NS	NSML	CS	CFCS	Noonan‐like	*P* value
LVEDD (mm), median (25–75th centile)	23.2 (18.6–30.9)	23.2 (18.6–31)	24.9 (18.4–29)	20.1 (18.8–21)	19.1 (19–19.2)	26.2 (20.8–33.6)	0.489
LVEDD *z* score, mean (SD)	−1 (0.97)	−1.57 (0.9)	−2.36 (1.2)	−3.21 (3.1)	−3.2 (0.9)	+5.5 (0.7)	**0.039**
LA diameter (mm), median (25–75th centile)	25.7 (18.3–30.9)	23 (15.2–30.5)	29 (25.8–42)	—	—	25.6 (19.6–29)	0.309
LA diameter *z* score, mean (SD)	+19 (3.2)	+19.9 (3.5)	—	—	—	+20.6	0.969
MLVWT (mm), median (25–75th centile)	11 (8–14)	11 (9–14)	13.5 (10–15.5)	7.5 (7–8.4)	8.2 (5–8)	7 (6–12.5)	**0.004**
MLVWT *z* score, mean (SD)	+9.6 (1.9)	+9.9 (2.1)	+17 (8.7)	+7 (2.1)	+6.4 (3.1)	+6.5 (5)	**0.074**
LVOT gradient (mmHg), median (25–75th centile)	23 (8–60)	20 (9–60)	60 (36–80)	8 (4–45)	27 (5–32)	6 (4–10)	**0.004**
LVOTO, *n* (%)	44 (39.1)	32 (28.9)	8 (66.7)	1 (16.7)	2 (33.3)	1 (9.1)	**0.032**
Mid‐cavity obstruction, *n* (%)	36 (24.2)	28 (25.2)	6 (50)	—	1 (16.67)	1 (9.1)	**0.009**
SAM, *n* (%)	44 (29.5)	33 (29.7)	8 (66.7)	1 (16.7)	—	2 (18.2)	**0.012**
RVH, *n* (%)	46 (48.9)	33 (63.5)	6 (66.7)	1 (16.7)	1 (16.67)	4 (36.4)	0.287
RVOT gradient (mmHg), median (25–75th centile)	10 (4–30)	10 (4–27)	5 (1–30)	2 (2–2.5)	2 (—)	4 (2.5–17)	**0.019**
RVOTO, *n* (%)	18 (39.1)	14 (16.2)	3 (25)	2 (33.3)	—	1 (9.1)	0.607
EF (%), median (25–75th centile)	79 (73–85)	77 (72–85)	81	83.5 (81–86)	89 (—)	77 (74.5–79.5)	0.871
Systolic dysfunction, *n* (%)	1 (3)	1 (3)	—	—	—	—	0.631
E/E′ average, median (25–75th centile)	10.77 (7.4–15.1)	10.9 (7.3–15.3)	10 (9.6–12.8)	10.2 (9.6–11.6)	8.6 (—)	—	0.183
Diastolic dysfunction, *n* (%)	9 (30)	8 (7.2)	1 (8.3)	—	—	—	0.456
ASH, *n* (%)	34 (26)	24 (21.6)	3 (25)	2 (33.3)	3 (50)	2 (16.7)	
Concentric, *n* (%)	52 (39.7)	33 (29.7)	7 (58.3)	2 (33.3)	3 (50)	7 (58.3)	
Eccentric, *n* (%)	4 (3.1)	4 (5.4)	—	—	—	—	
Apical, *n* (%)	3 (2.3)	3 (4.1)	—	—	—	—	
Unknown, *n* (%)	18 (12.1)	10 (9)	2 (16.7)	2 (33.3)	—	3 (25)	

ASH, Asymmetric septal hypertrophy; CFCS, cardiofaciocutaneous syndrome; CS, Costello syndrome; EF, ejection fraction; LA, left atrial; LVEDD, left ventricular end‐diastolic diameter; LVOT, left ventricular outflow tract; LVOTO, left ventricular outflow tract obstruction; MLVWT, maximal left ventricular wall thickness; NS, Noonan syndrome; NSML, Noonan syndrome with multiple lentigines; RVH, right ventricular hypertrophy; RVOT, right ventricular outflow tract; RVOTO, right ventricular outflow tract obstruction; SAM, systolic anterior motion of the mitral valve.Bold numbers represent statistically‐significant p values

### Electrocardiogram

Ninety‐three patients (62.4%) had electrocardiograms documented at baseline. Of those, 83 (89.2%) had one or more abnormal features. The majority (*N* = 91, 97.8%) were in sinus rhythm, with one patient having an atrial tachycardia and another being in junctional rhythm. Forty‐seven (59.5%) had QRS axis deviation, with 21 (44.7%) having a superior axis; 60 (69.8%) had criteria for LVH; and 30 (34.9%) had repolarization abnormalities in the form of T‐wave inversion in one or more location. The electrocardiographic data are summarized in Supporting Information, *Table* *S9*.

### Outcomes

**Figure 1:**
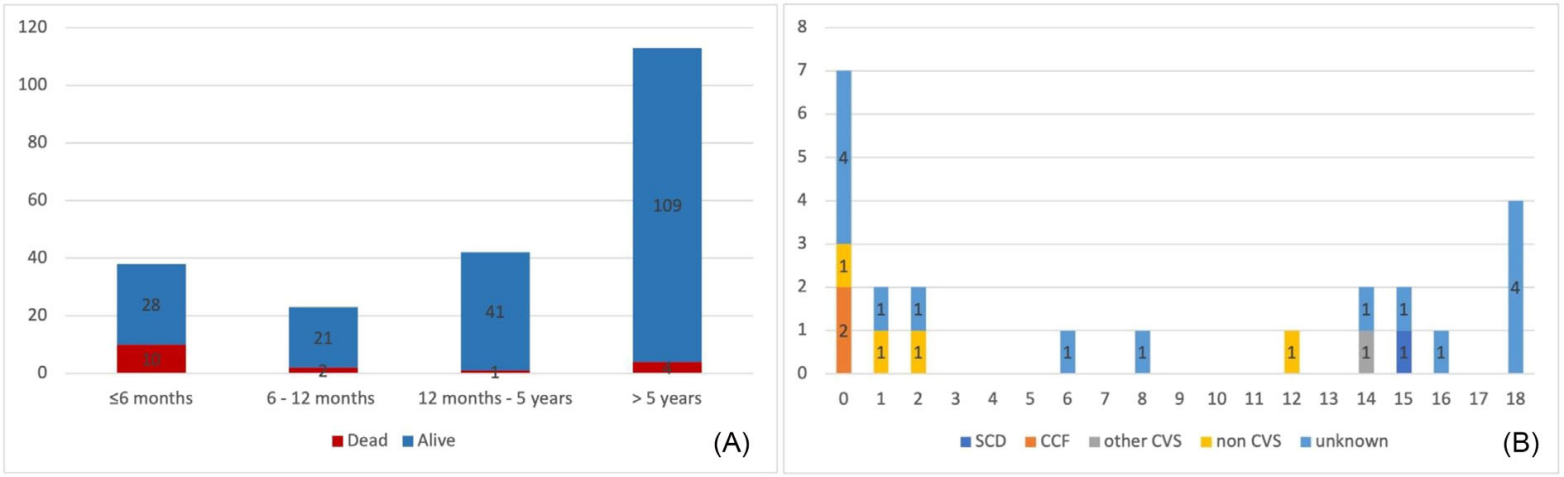
(A) Absolute number of deaths according to each age category. (B) Cause of death by age of death (years) [sudden cardiac death (SCD), congestive cardiac failure (CCF), and cardiovascular (CVS)].

The median length of follow‐up was 197.5 (IQR 93.58–370) months, or 231.55 patient‐months, with two patients (1.34%) lost to follow‐up. At the end of follow‐up, 126 patients (84.6%) were alive, including 14 (9.7%) who had undergone surgical myectomy (one of whom subsequently died with no documented cause of death available) and 3 (2%) who had undergone a heart transplant (one of whom subsequently died 14.2 years later with no documented cause of death available). Twelve patients (8.2%) had a major arrhythmic cardiac event (SCD or equivalent event) documented. A total of 23 patients (15.4%) died, at a median age of 24.1 (IQR 5.6–175.9) months. The cause of death was unknown in 12 cases (52.2%). Of the known causes, four patients died from a non‐CCF‐related CVS cause (17.4%) or from a non‐CVS‐related cause (17.4%). Two patients (8.7%) died due to progressive CCF, and one patient (4.4%) suffered an SCD (see *Figure* *1*). Seven patients with a past medical history of CCF (31.8%) and 11 patients presenting for first assessment under the age of 6 months (29%) died. A breakdown of outcomes by RASopathy syndrome is presented in *Table* *3*. There was no significant difference in survival or outcome by era of presentation or by genotype (Supporting Information, *Figure* *S4* and *Tables* *S4* and *S10*).

**Table 3:** Outcomes

	Total	NS	NSML	CS	CFCS	Noonan‐like	*P* value
Death, *n* (%)	21 (14.1)	13 (11.7)	1 (8.3)	1 (16.7)	1 (16.7)	3 (27.3)	**0.083**
SCD, *n* (%)	1 (4.8)	1 (7.7)	—	—	—	—	
CCF, *n* (%)	2 (9.5)	1 (7.7)	1 (8.3)	—	—	—	
Other CVS, *n* (%)	1 (4.8)	1 (7.7)	—	—	—	—	
Other, *n* (%)	4 (19.1)	2 (15.4)	—	1 (100)	—	1 (33.3)	
Unknown, *n* (%)	12 (57.1)	7 (53.9)	—	—	1 (100)	2 (66.7)	
Age at death (months), median (25–75th centile)	24.1 (5.6–175.9)	25.9 (5.6–175.9)	1.7	12.9	191.1	23.1 (12–73.8)	0.469
Myectomy, *n* (%)	14 (9.4)	13 (11.7)	1 (8.3)	—	—	—	0.886
ICD implantation, *n* (%)	7 (4.7)	7 (6.3)	—	—	—	—	0.653
CCF admission, *n* (%)	10 (6.7)	9 (8.1)	1 (8.3)	—	—	—	0.715
Heart transplant, *n* (%)	3 (2)	3 (2.7)	—	—	—	—	0.909
NSVT, *n* (%)	5 (3.4)	3 (3)	1 (8.3)	—	—	1 (33.3)	0.674
SCD or equivalent event, *n* (%)	12 (8.1)	9 (8.1)	1 (8.3)	—	—	2 (18.2)	0.775

CCF, congestive cardiac failure; CFCS, cardiofaciocutaneous syndrome; CS, Costello syndrome; CVS, cardiovascular; ICD, implantable cardiac defibrillator; *n*, number of patients; NS, Noonan syndrome; NSML, Noonan syndrome with multiple lentigines; NSVT, non‐sustained ventricular tachycardia; SCD, sudden cardiac death.Bold numbers represent statistically‐significant p values

### Survival and predictors of all‐cause mortality and sudden cardiac death or equivalent event

**Figure 2:**
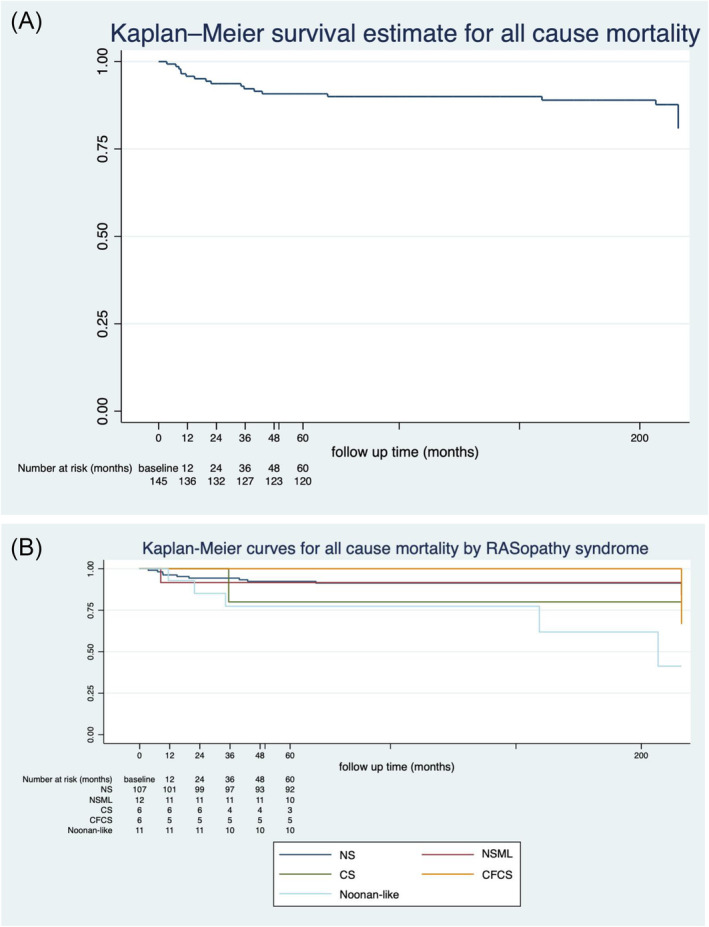
Kaplan–Meier curve for all‐cause mortality with yearly numbers at risk for (A) whole cohort and (B) by different RASopathy syndromes [Noonan syndrome (NS), Noonan syndrome with multiple lentigines (NSML), Costello syndrome (CS), and cardiofaciocutaneous syndrome (CFCS)].

**Figure 3:**
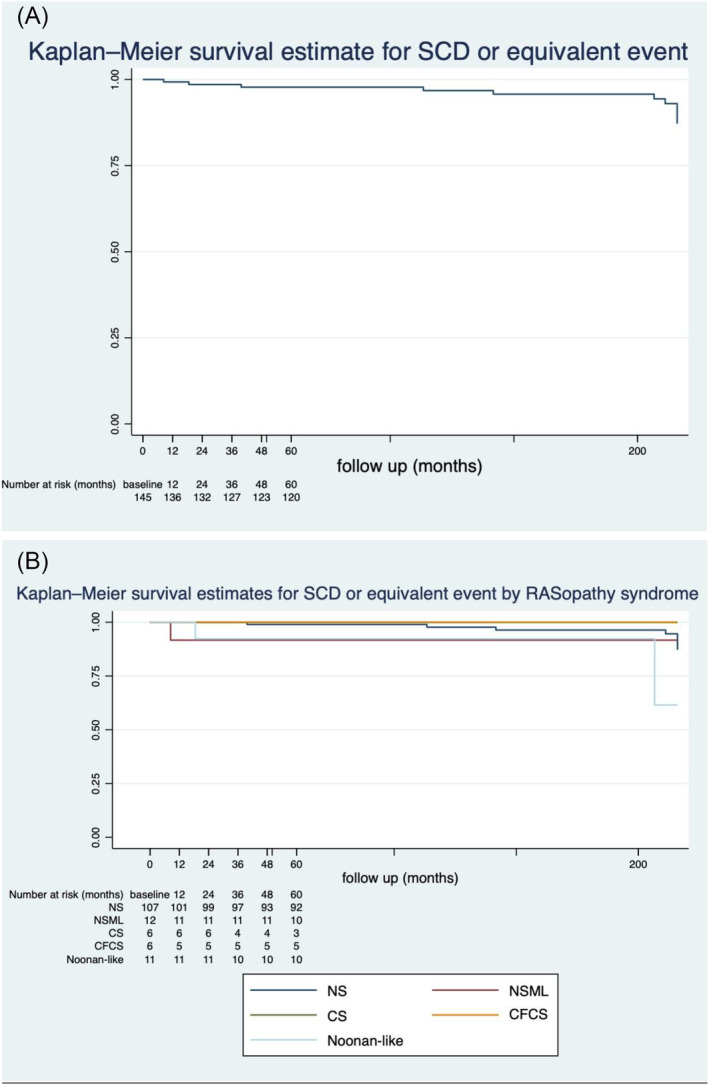
Kaplan–Meier curve for sudden cardiac death (SCD) or equivalent event with yearly numbers at risk for (A) whole cohort and (B) by different RASopathy syndromes [Noonan syndrome (NS), Noonan syndrome with multiple lentigines (NSML), Costello syndrome (CS), and cardiofaciocutaneous syndrome (CFCS)].

Overall survival was 96.45% [95% confidence interval (CI) 91.69–98.51], 90.42% (95% CI 84.04–94.33), and 84.12% (95% CI 75.42–89.94) at 1, 5, and 10 years, respectively, but this varied by RASopathy syndrome (*Table* *4* and *Figure* *2*). Symptoms at baseline assessment, presence of concomitant CHD, RASopathy syndrome, past medical history of CCF, CCF admission, presence of non‐sustained ventricular tachycardia (NSVT), and MLVWT were identified as predictors of all‐cause mortality on univariate analysis (*Table* *5*). Concerning SCD or equivalent event (*Figure* *3*), the presence of NSVT, past medical history of CCF, and LVOT gradient were identified as predictors on univariate analysis (see *Table* *6*).

**Table 4:** Survival

	1 year, % (95% CI)	5 years, % (95% CI)	10 years, % (95% CI)	15 years, % (95% CI)
NS	94.3 (87.7–97.4)	91.3 (83.9–95.4)	91.3 (83.9–95.4)	91.3 (83.9–95.4)
NSML	91.7 (53.9–98.8)	91.7 (53.9–98.8)	91.7 (53.9–98.8)	91.7 (53.9–98.8)
CS	81.8 (23.9–97.2)	81.8 (23.9–97.2)	81.8 (23.9–97.2)	81.8 (23.9–97.2)
CFCS	100 (—)	100 (—)	50 (0.6–91.1)	50 (0.6–91.1)
Noonan‐like	82.9 (47.2–95.5)	73.7 (32.8–83.3)	58.9 (32.8–83.3)	39.3 (7–72)

CFCS, cardiofaciocutaneous syndrome; CI, confidence interval; CS, Costello syndrome; NS, Noonan syndrome; NSML, Noonan syndrome with multiple lentigines.

**Table 5:** Predictors of all‐cause mortality

	Hazard ratio	Standard error	95% CI	*P* value
Demographics and baseline clinical characteristics				
Gender	0.83	0.38	0.33–2.05	0.679
Age at diagnosis	1	0.01	0.99–1.01	0.864
Age at baseline assessment	0.99	0.01	0.98–1	0.102
Proband	2.57E + 14	3.72E + 21	—	1
FHx HCM	1.37E − 15	2.10E − 08	—	1
PMHx CHD	2.32	1.09	0.92–5.86	**0.073**
PMHx CCF	0.45	0.21	0.18–1.14	**0.092**
PMHx arrhythmia	1.13	1.17	0.15–8.54	0.906
Symptoms	1.31	0.59	0.54–3.17	**0.017**
Medications	0.98	0.43	0.41–2.31	0.967
CCF admission	4.31	2.4	1.45–12.83	**0.009**
NSVT	5.56	4.3	1.22–25.35	**0.027**
Syndrome				**0.011**
NSML	0.68	0.71	0.09–5.22	0.714
CS	1.6	1.67	0.21–12.27	0.65
CFCS	1.46	1.52	0.019–11.16	0.715
Noonan‐like	3.81	2.02	1.35–10.79	**0.012**
Gene	1.02	0.69	0.27–3.82	0.22
Echocardiographic phenotype				
LVEDD	0.956	0.36	0.89–1.03	0.225
LVEDD *z* score	1.02	0.04	0.95–1.1	0.533
LA diameter	0.99	0.52	0.89–1.1	0.825
LA diameter *z* score	1.02	0.06	0.91–1.14	0.784
MLVWT	0.85	0.07	0.73–0.99	**0.044**
MLVWT *z* score	0.97	0.04	0.9–1.06	0.538
LVOT gradient	0.99	0.01	0.97–1.01	0.318
RVOT gradient	0.99	0.02	0.96–1.02	0.625
Ejection fraction	1.08	0.07	0.95–1.23	0.223
Average E/E′	0.97	0.09	0.81–1.16	0.711
RVH	0.49	0.27	0.16–1.57	0.202
Mid‐cavity obstruction	1.56	0.87	0.52–4.68	0.428
SAM	0.68	0.37	0.24–1.96	0.478

CCF, congestive cardiac failure; CFCS, cardiofaciocutaneous syndrome; CHD, congenital heart defect; CI, confidence interval; CS, Costello syndrome; FHx, family history; HCM, hypertrophic cardiomyopathy; LA, left atrial; LVEDD, left ventricular end‐diastolic diameter; LVOT, left ventricular outflow tract; MLVWT, maximal left ventricular wall thickness; NSML, Noonan syndrome with multiple lentigines; NSVT, non‐sustained ventricular tachycardia; PMHx, past medical history; RVH, right ventricular hypertrophy; RVOT, right ventricular outflow tract; SAM, systolic anterior motion of the mitral valve.Bold numbers represent statistically‐significant p values

**Table 6:** Predictors of SCD or equivalent event

	Hazard ratio	Standard error	95% CI	*P* value
Demographics and baseline clinical characteristics				
Gender	1.47	0.89	0.45–4.84	0.522
Age at diagnosis	1	0.01	0.99–1.02	0.556
Age at baseline assessment	0.99	0.01	0.98–1.01	0.506
Proband	0.57	0.60	0.07–4.5	0.598
FHx HCM	0.63	0.66	0.08–4.93	0.659
PMHx CHD	1.65	1.11	0.44–6.15	0.457
PMHx CCF	0.34	0.23	0.09–1.26	0.096
PMHx arrhythmia	6.42E + 14	2.30E + 22	—	1.000
Symptoms	1.53	0.96	0.45–5.25	0.497
Medications	0.48	0.30	0.14–1.64	0.243
CCF admission	1.75	1.83	0.22–13.68	0.596
NSVT	6.1	4.84	1.28–28.91	**0.023**
Syndrome				0.514
NSML	1.11	1.18	0.14–8.88	0.921
CS	5.08E − 16	3.88E − 08	—	1.000
CFCS	5.09E − 16	3.45E − 08	—	1.000
Noonan‐like	3.07	2.45	0.64–14.6	0.159
Gene	1.24	1.04	0.24–6.41	0.82
Gene negative	1.81	1.22	0.49–6.75	0.376
Echocardiographic phenotype				
LVEDD	0.87	0.80	0.74–1.04	0.126
LVEDD *z* score	0.64	0.17	0.38–1.08	0.106
LA diameter	0.96	0.08	0.81–1.14	0.657
LA diameter *z* score	0.99	0.09	0.82–1.19	0.893
MLVWT	1.00	0.07	0.88–1.15	0.944
MLVWT *z* score	1.01	0.03	0.95–1.08	0.783
LVOT gradient	1.02	0.01	1–1.04	**0.031**
RVOT gradient	1.02	0.02	0.99–1.06	0.186
Ejection fraction	1.03	0.09	0.86–1.24	0.726
Average E/E′	5.09E − 16	3.45E − 08	—	1.000
RVH	0.43	0.37	0.08–2.36	0.332
Mid‐cavity obstruction	0.70	0.54	0.16–3.16	0.647
SAM	0.67	0.51	0.15–2.97	0.597

CCF, congestive cardiac failure; CFCS, cardiofaciocutaneous syndrome; CHD, congenital heart defect; CI, confidence interval; CS, Costello syndrome; FHx, family history; HCM, hypertrophic cardiomyopathy; LA, left atrial; LVEDD, left ventricular end‐diastolic diameter; LVOT, left ventricular outflow tract; MLVWT, maximal left ventricular wall thickness; NSML, Noonan syndrome with multiple lentigines; NSVT, non‐sustained ventricular tachycardia; PMHx, past medical history; RVH, right ventricular hypertrophy; RVOT, right ventricular outflow tract; SAM, systolic anterior motion of the mitral valve; SCD, sudden cardiac death.Bold numbers represent statistically‐significant p values

## Discussion

This UK and Ireland cohort study is, to our knowledge, the largest description of the natural history of RASopathy‐associated HCM. The major findings are the demonstration of phenotypic differences according to the underlying RASopathy syndrome, the description of a discrete group of patients with Noonan‐like syndrome and a distinct cardiac phenotype with overall worse survival, and the identification of potential predictors for all‐cause mortality and SCD or equivalent event.

### Presentation and cardiac phenotype

Large registry studies of paediatric HCM have provided valuable information regarding the long‐term prognosis of patients with sarcomeric and non‐syndromic HCM,^4^, ^32^, ^33^, ^34^ but the data are more limited for non‐sarcomeric aetiologies. In keeping with previous reports,^35^, ^36^, ^37^ this study confirms that onset of HCM in individuals with RASopathy‐related HCM usually occurs during infancy, significantly younger than that of sarcomeric HCM. Our study also confirms the importance of additional cardiac ‘red flags’ that should trigger consideration of a RASopathy syndrome as the cause of HCM in young children, including the presence of coexisting CHD, concomitant RVH and RVOTO, and extreme QRS axis deviation, in keeping with previous studies^21^, ^35^, ^36^, ^38^, ^39^ and as suggested by the recently published European Society of Cardiology (ESC) guidelines for the management of cardiomyopathies.^40^ Although patients with RASopathy syndromes most commonly do not have a family history HCM,^14^, ^41^ familial HCM was present in a significant minority of patients in our cohort, highlighting the importance of a thorough family history and examination, even in children with syndromic disease.

### Correlation of clinical syndrome and genotype with cardiac phenotype

A major strength of this study is the high frequency of genetic testing and diagnostic yield, allowing genotype–phenotype correlations to be explored. The proportion of patients undergoing genetic testing and the subsequent yield of testing increased significantly over time, reflecting advances in genetic knowledge, and changing clinical practice. It is possible, therefore, that more robust genotypes–phenotypes may exist than we have been able to demonstrate. Patients with variants in *PTPN11* and *RIT1* were diagnosed with HCM at a younger age; this may be related to the fact that CHD was also more common with these genotypes, as the suspicion of CHD may have prompted earlier investigation and an echocardiographic diagnosis of HCM. While the cardiac phenotype was otherwise largely similar across the different clinical syndromes, patients with NSML had the most severe LVH and the highest resting LVOT gradients, while patients with CS and CFCS had lower maximal LV wall thicknesses and were less likely to have resting LVOTO. Similarly, patients with variants in *PTPN11* and *RAF1* had higher MLVWT and resting LVOT gradients as well as higher likelihood of mid‐cavity obstruction, while those with *HRAS* variants had less LVH and a lower prevalence of resting LVOTO. This is in keeping with previous studies that have shown particularly severe cardiac phenotypes in children with NSML^15^ and has implications for consideration of novel treatments such as MEK inhibitors, which have shown some promise in the treatment of severe HCM in infants with NS and NSML,^42^, ^43^ as recognized by recent guidance.^40^, ^44^

A novel finding in this study is the identification of a distinct group of patients with a clinical diagnosis of Noonan‐like syndrome, 50% of whom had a variant in a RASopathy gene that was either a VUS or did not fit the clinical syndrome described in the literature, whose features did not fit into one of the other RASopathy syndrome categories. Although their demographics and baseline clinical characteristics were similar to those of the other RASopathy syndromes, they did have a significantly higher prevalence of extra‐cardiac manifestations. The cardiac phenotype was less severe than other RASopathy syndromes, with less significant LVH and no resting LVOTO. However, mortality was high, with a 5 year survival of <60%. Although these data should be interpreted with caution, given the small numbers of patients and the fact that the cause of death was unknown in four out of five patients (and non‐cardiac in the remaining patient), the findings suggest that it is important to identify this group of patients with apparently mild HCM who nevertheless have a significantly poorer outcome than other RASopathy syndromes. Given the higher prevalence of extra‐cardiac manifestations in this subgroup, it is possible that non‐cardiac causes of death may predominate in patient with Noonan‐like syndrome.

### Survival and predictors of outcome

Survival in patients with RASopathy‐related HCM is highly dependent on age at diagnosis,^34^, ^36^ a finding confirmed in this study. CCF has been reported as the most common cause of cardiac‐related death in RASopathy‐associated HCM.^17^, ^36^, ^41^ This was not confirmed in our study, although it is possible that CCF‐related deaths are underestimated as the cause of death was unknown in half of our cohort. In keeping with previous studies,^36^, ^45^ CHD, history of CCF prior to baseline presentation, and CCF requiring admission to hospital were predictors of all‐cause mortality on univariate analysis in our cohort. Symptoms at baseline, NSVT, and MLVWT have all been shown to be predictors of mortality in large registry studies for HCM in children^4^, ^46^, ^47^ and are now correlated with RASopathy‐associated HCM specifically. Importantly, we have shown for the first time that the underlying RASopathy syndrome is an additional potential risk factor for mortality, likely driven by the cohort of patients with Noonan‐like syndrome. These findings highlight the importance of the underlying diagnosis in the clinical management of RASopathy patients. Further large international studies would allow for higher event numbers to further explore independent predictors of all‐cause mortality in this population.

Arrhythmic adverse events are rarely described in patients with RASopathy‐associated HCM, with reported frequencies of ventricular arrhythmias of <2%.^17^, ^45^, ^48^, ^49^ The results of our study suggest that this may be a significant underestimate; nearly 5% of our cohort had a VT or VF episode, which is more in line with a recent, large (*n* = 188), international, multicentre study.^50^ These findings highlight the importance of considering ventricular arrhythmia and sudden death risk in individuals with RASopathy syndromes. There are currently no established guidelines for assessing ventricular arrhythmia risk in patients with RASopathy syndromes, and it is not known whether risk stratification algorithms for non‐syndromic HCM^51^, ^52^ are also applicable to patients with RASopathy syndromes. However, the finding in our study that there are potential predictors for SCD or equivalent event suggests that specific risk factors for ventricular arrhythmia in patients with RASopathy syndromes may be present. Importantly, one of the predictors highlighted in the univariate analysis, LVOT gradient, is potentially modifiable, which may have implications for the treatment of obstructive HCM in this population, even in the absence of symptoms. Future studies to identify RASopathy‐specific risk factors for ventricular arrhythmia will be important to address this unmet need.

### Limitations

This study is limited by inherent problems of retrospective studies, in particular missing or incomplete data. Variations in clinical assessment and patient management are inevitable as patients were recruited from multiple centres and across different eras. Genetic testing was performed at the participating clinicians' discretion. Although a high proportion of patients with a RASopathy syndrome had a disease‐causing variant identified on genetic testing, it is not known whether genetic testing results altered the final diagnosis or confirmed previous clinical suspicions. The exact number of patients who had additional genetic testing with a cardiomyopathy panel is not available due to the retrospective nature of the study, and therefore, an extrapolation on the prevalence of a sarcomeric variant coexisting in this cohort could not be determined. Variations in echocardiographic protocols and availability of images for retrospective assessment in different centres and eras resulted in missing variables. A strict cut‐off value of E/E′ > 14 used to define diastolic dysfunction may have resulted in missing patients with a suspicion of elevated filling pressures with an E/E′ of 10–14. Although the mortality rate is unlikely to be affected by these missing data, other phenotypic features or outcomes could have been underestimated or overestimated. Cause of death was not documented in a substantial proportion of cases, making conclusions regarding this subject challenging. Mortality and SCD or equivalent event were rare events; thus, a multivariate analysis of could not be performed. Data collection for this cohort relied on patients being referred to collaborating paediatric cardiology centres. Therefore, it is possible that patients who either had a very mild phenotype, not warranting referral to an expert centre, or, conversely, had a very severe phenotype resulting in early death in a neonatal or paediatric unit may not have been included in this study.

## Conclusions

To our knowledge, this is the largest cohort of RASopathy‐associated HCM that includes different RASopathy syndromes and genes. The findings show a heterogeneous clinical presentation, with differing phenotypes and outcomes according to underlying syndrome. This was most notable in a distinct category of patients with Noonan‐like syndrome who had a milder HCM phenotype but significantly worse survival. Potential predictors of all‐cause mortality and SCD or equivalent event for this population exist, but larger studies are required to further explore their significance.

## Conflict of interest

None declared.

## Funding

This work was supported by the Alexander S. Onassis Public Benefit Foundation. E.F. and J.P.K. are supported by Max's Foundation and Great Ormond Street Hospital Children's Charity. J.P.K. is supported by a Medical Research Council (MRC) Clinical Academic Research Partnership (CARP) award (MR/T024062/1).

## Supporting information


**Table S1:** Collaborating centres with corresponding patient numbers.
**Table S2:** Congenital heart defects by RASopathy syndrome.
**Table S3:** Patients with Noonan like syndrome.
**Table S4:** Patients with Noonan like syndrome with loose anagen hair.
**Table S5:** Demographics and baseline clinical characteristics by most prevalent genes.
**Table S6:** Clinical and genetics characteristics and outcomes by era of presentation.
**Table S7:** nucleotide and protein changes.
**Table S8:** Echocardiographic data by most prevalent genes.
**Table S9:** Electrocardiographic data at baseline assessment.
**Table S10:** Outcomes by most prevalent genes.
**Figure S1:** Extra‐cardiac manifestations by RASopathy syndrome X axis represents the absolute number of patients in each category by RASopathy syndrome and Y axis represents the system involved.
**Figure S2:** Age category by RASopathy Syndrome X axis represents the different RASopathy syndromes and Y axis represents the absolute number in each age category.
**Figure S3:** Gene mutation by RASopathy Syndrome X axis represents the absolute number of each RASopathy syndrome by gene variant and Y axis represents each RASopathy syndrome.
**Figure S4:** Kaplan–Meier survival estimates by era of presentation (x) axis represents frequencies and (y) axis analysis time in months, 95% confidence intervals are shown for the corresponding curves in shading, (*P* = 0.453).
