# Supplementary material for: Natural history and outcomes in paediatric RASopathy‐associated hypertrophic cardiomyopathy
Source: ESC Heart Fail. 2024 Jan 13;11(2):923–36. doi: 10.1002/ehf2.14637 (PMC10966228; doi:10.1002/ehf2.14637)
Supplement: Supplementary file 1 — Table S1: Collaborating centres with corresponding patient numbers. Table S2: Congenital heart defects by RASopathy syndrome. Table S3: Patients with Noonan like syndrome. Table S4: Patients with Noonan like syndrome with loose anagen hair. Table S5: Demographics and baseline clinical characteristics by most prevalent genes. Table S6: Clinical and genetics characteristics and outcomes by era of presentation. Table S7: nucleotide and protein changes. Table S8: Echocardiographic data by most prevalent genes. Table S9: Electrocardiographic data at baseline assessment. Table S10: Outcomes by most prevalent genes. Figure S1: Extra‐cardiac manifestations by RASopathy syndrome X axis represents the absolute number of patients in each category by RASopathy syndrome and Y axis represents the system involved. Figure S2: Age category by RASopathy Syndrome X axis represents the different RASopathy syndromes and Y axis represents the absolute number in each age category. Figure S3: Gene mutation by RASopathy Syndrome X axis represents the absolute number of each RASopathy syndrome by gene variant and Y axis represents each RASopathy syndrome. Figure S4: Kaplan–Meier survival estimates by era of presentation (x) axis represents frequencies and (y) axis analysis time in months, 95% confidence intervals are shown for the corresponding curves in shading, (P = 0.453). [file EHF2-11-923-s001.pdf]

Table S1: Collaborating centres with corresponding patient numbers

Centre	Number of patients*
Great Ormond Street Hospital, London	102
Bristol Royal Hospital for Children	15 (7 & 8)
Birmingham Children's Hospital	12 (9 & 3)
University Hospital of Wales, Cardiff	12 (8 & 4)
Royal Brompton Hospital, London	11 (6 & 5)
Glenfield Hospital, Leicester	8 (2 & 6)
Royal Hospital for Children, Glasgow	8 (2 & 6)
Evelina Children's Hospital, London	6 (2 & 4)
Southampton General Hospital	5 (2 & 3)
Alder Hey, Liverpool	3 (2 & 1)
Freeman's Hospital, Newcastle	2 (2 & 0)
Leeds General Infirmary	2 (2 & 0)
Our Lady's Children's Hospital, Dublin	2 (2 & 0)
John Radcliffe Hospital, Oxford	1 (1 & 0)

*The numbers add up to more than the total number of patients in this study – this is because some patients were seen in the local paediatric cardiology centre as well as Great Ormond Street Hospital as a national reference centre and were not included twice in the study numbers. In the parenthesis there is the breakdown of numbers, first number is patients only seen at the local centre, second number is patients seen in both the local and reference centre

Table S2: Congenital heart defects by RASopathy syndrome

	Total	NS	NSML	CS	CFCS	Noonan-like
ASD	16 (14.6)	13 (17.8)	-	-	1 (16.7)	2 (14.3)
VSD	8 (7.3)	5 (6.9)	-	-	1 (16.7)	-
PVS	33 (30)	22 (30.1)	2 (16.7)	2 (33.3)	4 (66.7)	3 (21.4)
PDA	5 (4.6)	3 (4.1)	1 (8.3)	-	-	1 (7.1)
Dysplastic valve	19 (17.3)	14 (19.2)	-	-	1 (16.7)	2 (14.3)
Polyvalvulopathy	21 (19.1)	16 (21.9)	1 (8.3)	1 (16.7)	1 (16.7)	2 (14.3)
AS	4 (3.6)	3 (4.1)	-	-	1 (16.7)	-
Other	6 (5.5)	5 (6.9)	-	-	-	-
>1	32 (29.1)	25 (34.3)	-	1 (16.7)	3 (50)	3 (21.4)
None	45 (40.9)	24 (32.9)	8 (66.7)	-	2 (33.3)	8 (57.1)
Unknown	39 (26.2)	38 (34.2)	-	1 (16.7)	-	-

NS: Noonan syndrome, NSML: Noonan syndrome with multiple lentigines, CS: Costello syndrome, CFCS: cardiofaciocutaneous syndrome, ASD: atrial septal defect, VSD: ventricular septal defect, PVS: pulmonary valve stenosis, PDA: patent ductus arteriosus, AS: aortic valve stenosis

Table S3: Patients with Noonan like syndrome

Patient number	1	2	3	4	5	6	7	8	9	10	11
Baseline demographics and clinical characteristics											
Gender	M	F	F	M	M	F	M	F	M	F	M
Proband?	Yes	Yes	-	-	-	Yes	Yes	Yes	Yes	Yes	-
Age at diagnosis (months)	4.9	121.9	-	133.4	135.2	0.4	-	2.6	1.3	1.2	-
Age at baseline (months)	29.4	63.8	7.7	133.4	135.2	0.43	0.53	1.15	0.8	11.9	16.3
PMHx CHD	No	Yes	No	No	No	Yes	Yes	Yes	No	No	No
Extra-cardiac manifestations	Yes	Yes	No	No	No	No	Yes	Yes	No	No	-
PMHx CCF	No	No	-	No	No	No	No	Yes	Yes	No	-
Symptoms	No	No	No	No	No	No	No	Yes	Yes	No	No
Medications	No	Yes	No	No	No	Yes	No	Yes	Yes	Yes	No
Outcomes											
Follow up (months)	216	206.7	216	159.33	216	100.1	102.8	11.4	18.6	94.3	144.9
Death	No	Yes	No	Yes	No	No	No	Yes	No	No	No
Cause of death	-	Non CVS	-	Unknown	-	-	-	Unknown	-	-	-
Age at death (months)	-	12	-	191.2	-	-	-	2.4	-	-	-
SCD or equivalent event	No	Yes	No	No	No	No	No	No	Yes	No	No
CCF Admission	No	No	No	No	No	No	No	No	No	No	No
Myectomy	No	No	No	No	No	No	No	No	No	No	No
Echocardiographic parameters											
LVEDD (mm)	-	25.7	-	-	36.1	26.6	32.2	14	-	17.2	-
LVEDD z score	-	-2.4	-	-	2.6	1.9	5.5	-4.3	-	-3.2	-
LA diametre (mm)	-	-	-	-	29	-	25.6	-	-	19.6	-
LA diametre z score	-	-	-	-	-	-	20.6	-	-	15	-
MLVWT (mm)	-	6	-	10	9	6	7	-	15	-	-
MLVWT z score	-	3.4	-	-	3.2	4.3	6.5	-	-	-	-
LVOT gradient (mmHg)	-	-	-	4	-	5	10	10	117	-	-
LVOTO	-	-	-	No	-	No	No	No	Yes	No	-
Mid cavity obstruction	-	No	-	No	No	No	No	No	Yes	No	-

SAM	-	No	-	No	No	No	No	Yes	Yes	No	-
RVH	-	No	-	No	No	Yes	Yes	-	Yes	Yes	-
RVOT gradient (mmHg)	-	-	-	-	1	-	4	-	30	-	-
RVOTO	-	-	-	-	No	-	No	-	Yes	-	-
EF (%)	-	-	-	-	-	79	80	-	-	74	-

PMHx: past medical history, CCF: congestive cardiac failure, CHD: congenital heart defects, ICD: implantable cardiac defibrillator, SCD: sudden cardiac death, LVEDD: left ventricular end diastolic diameter, MLVWT: maximal wall thickness, LAd: left atrial diameter, LVOT: left ventricular outflow tract, LVOTO: LVOT obstruction, SAM: systolic anterior motion of the mitral valve, RVH: right ventricular hypertrophy, RVOT: right ventricular outflow tract, RVOTO: RVOT obstruction, EF: ejection fraction

Table S4: Patients with Noonan like syndrome with loose anagen hair

	Patient 1	Patient 2	Patient 3
Baseline demographics and clinical characteristics			
Gender	Male	Male	Male
Proband?	Yes	Yes	Yes
Age at diagnosis (months)	81.3	-	-
Age at baseline (months)	67.7	64	6.5
PMHx CHD	No	Yes	No
Extra-cardiac manifestations	No	No	No
PMHx CCF	No	No	No
Symptoms	No	No	No
Medication	No	b-blockers	No
Outcomes			
Follow up (months)	198.9	9.8	16.6
Death	No	Yes	Yes
Cause of death	-	Unknown	Unknown
Age at death (months)	-	73.8	23.1
SCD or equivalent event	No	No	No
Myectomy	No	No	No
CCF admission	No	No	No
ICD implantation	No	No	No
Heart transplant	No	No	No
Echocardiographic parameters			
LVEDD (mm)	29.7	-	-
LVEDD z score	+4.7	-	-
LA diametre (mm)	26	25	
LA diametre z score	+3.4	-	-
MLVWT (mm)	8	7	9
MLVWT z score	+9.2	-	-
LVOT gradient (mmHg)	45	16	27
LVOTO	Yes	No	No
Mid cavity obstruction	No	No	No
SAM	No	No	No
RVH	Yes	No	No
RVOT gradient (mmHg)	-	-	4
RVOTO	No	No	No
EF (%)	75	-	-
Systolic dysfunction	No	-	-

PMHx: past medical history, CCF: congestive cardiac failure, CHD: congenital heart defects, ICD: implantable cardiac defibrillator, SCD: sudden cardiac death, LVEDD: left ventricular end diastolic diameter, MLVWT: maximal wall thickness, LAd: left atrial diameter, LVOT: left ventricular outflow tract, LVOTO: LVOT obstruction, SAM: systolic anterior motion of the mitral valve,

RVH: right ventricular hypertrophy, RVOT: right ventricular outflow tract, RVOTO: RVOT obstruction, EF: ejection fraction

Table S5: Demographics and baseline clinical characteristics by most prevalent genes

	PTPN11	RAF1	RIT1	HRAS	p value
Gender (Male), n (%)	16 (55.2)	12 (66.7)	6 (75)	3 (37.5)	0.431
Age at diagnosis (months), median (25th-75th centile)	0.4 (0 - 9)	2.7 (0.1 - 8.4)	0.23 (0 - 8.7)	2.83 (0 - 121.9)	**0.041**
Age at baseline (months), median (25th-75th centile)	11.1 (5.7 - 50.8)	37.6 (11.6 - 64.3)	2.41 (0.11 - 8.8)	12.1 (6.7 - 35.4)	0.889
Proband, n (%)	22 (75.9)	16 (88.9)	7 (87.5)	8 (100)	0.741
FHx HCM, n(%)	5 (17.2)	1 (5.6)	-	-	0.472
PMHx CCF, n(%)	10 (34.5)	2 (11.1)	-	-	0.151
PMHx arrhythmia, n (%)	3 (10.3)	1 (5.6)	1 (12.5)	1 (12.5)	1
CHD, n (%)	16 (55.2)	3 (16.7)	7 (87.5)	2 (25)	**0.002**
Extra-cardiac manifestations	10 (34.5)	10 (55.6)	2 (25)	3 (37.5)	0.531
Symptoms, n (%)	10 (34.5)	8 (44.4)	2 (25)	-	0.143
Medications, n (%)	18 (62.1)	11 (61.1)	4 (57.1)	4 (50)	0.958

n: number of patients, FHx: family history, HCM: hypertrophic cardiomyopathy, SCD: sudden cardiac death, PMHx: past medical history, CCF: congestive cardiac failure, CHD: congenital heart defects

Table S6: Clinical and genetics characteristics and outcomes by era of presentation

	1985-1999	2000-2010	2011-2020	p value (*)	p value (**)
	(n = 18)	(n = 56)	(n = 75)		
Male, n (%)	10 (55.6)	36 (64.3)	47 (62.7)	0.708	0.758
Median age at presentation, months (IQR)	89.7 (29.6 – 139.7)	323 (8.7 – 92.1)	11.9 (2.7 – 61.8)	0.003	0.063
Syndrome, n (%)				0.124	0.152
NS	15 (83.3)	43 (76.8)	53 (70.7)		
NSML	3 (16.7)	2 (3.6)	7 (9.3)		
CS		1 (1.8)	5 (6.7)		
CFCS		5 (8.9)	1 (1.4)		
Noonan-like		4 (7.1)	8 (10.7)		
NS_LAH			3 (4)		
Genetic testing done, n (%)	9 (50)	44 (78.6)	64 (85.3)	<0.001	<0.001
Variant identified (yes), n (%)	3 (33.3)	27 (61.4)	50 (78.1)	<0.001	0.007
Which variant, n (%)	PTPN11 2 (66.7)	PTPN11 7 (25.9)	PTPN11 20 (40)	0.255	0.095
	KRAS 1 (33.3)	RAF1 6 (22.2)	RAF1 12 (24)		
		RIT1 4 (14.8)	RIT1 4 (8)		
		HRAS 2 (7.4)	HRAS 6 (12)		
		KRAS 1 (3.7)	KRAS 4 (8)		
		LZTR1 4 (14.8)	BRAF 1 (2)		
		BRAF 2 (7.4)	SHOC2 3 (6)		
		MEK2 1 (3.7)			
2nd variant identified (yes), n (%)	-	4 (7.1)	1 (1.3)	0.742	0.365
Median follow up, months (IQR)	209.5 (167.4 – 216)	215.7 (215 – 216)	113.1 (43.9 – 182.9)	<0.001	<0.001

SCD or equivalent event, n (%)		2 (11.1)	4 (7.1)	6 (8)	0.457	0.959
Heart transplant, n (%)		-	1 (1.8)	2 (2.7)	0.197	0.829
Myectomy, n (%)		3 (16.7)	5 (8.9)	6 (8)	0.447	0.405
Death, n (%)		3 (16.7)	8 (14.3)	12 (16)	0.62	0.453

n: number of patients, IQR: interquartile range, NS: Noonan syndrome, NSML: Noonan syndrome with multiple lentigines, CS: Costello syndrome, CFCS: cardiofaciocutaneous syndrome, SCD: sudden cardiac death. (*) represents p values for whole group, (**) subgroup analysis excluding the first era

Table S7: nucleotide and protein changes

Affected Gene	Nucleotide code	Protein code	N
PTPN11	836A>G	Tyr279Cys	6
	1528C>G	Gln510Glu	3
	922A>G	Asn308Asp	2
	102G>T	Lys34Asn	1
	188A>G	Tyr63Cys	1
	846C>G	Ile282Met	1
	218C>T	Thr73Ile	1
	923A>G	Asn308Ser	1
	854T>C	Phe285Ser	1
	236A>G	Glu79Arg	1
	417G>C	Glu139Asp	1
	1528C>G	Gln510Glu	1
	768G>C	Asn320Ser	1
	1403C>T	Thr468Met	1
RAF1	770C>T	Ser257Leu	2
	770C>T	Ser257Gly	1
	766A>G	Arg256Gly	1
	775T>A	Ser259Thr	1
	1082G>C	Gly361Ala	1
	779c >T	Thr260lle	1
	76BG>T	Arg256Ser	1
	781C>T	Pro261Ser	1
RIT1	244T>C	Phe82Leu	2
	151G>T	Asp51Tyr	1
	284G>C	Gly95Ala	1
	229G>A	Ala77Thr	1
	244T>A	He82Lle	1
HRAS	34G>A	Gly12Ser	5
	64C>A	Gln22Lys	1
	466T>C	Phe156Leu	1
	34G>T	Gly12Cys	1
KRAS	179G>T	Gly60Val	2
	346A>C	ASn116His	1
	173C>T	Thr58IIe	1
LZTR1	3493C>T	Lys1165Glu	1
	1234C>T	Arg412Cys	1
	290G>T	Arg97Leu	1
SHOC2	4A>G	Ser2Gly	1
BRAF	1782T>G	Asp5974Glu	1

MEK2	619G>A	Glu207Lys	1

Table S8: Echocardiographic data by most prevalent genes

	PTPN11	RAF1	RIT1	HRAS	p-values
LVEDD (mm), median (25-75th centile)	23.8 (20.2 - 29)	23.3 (20 - 31.5)	18.1 (17.5 - 19.6)	21 (17.2 - 26.6)	0.469
LVEDD z score, mean (SD)	+0.01 (2.6)	-0.15 (3)	-2.66 (0.9)	-	0.565
LA diametre (mm), median (25-75th centile)	27 (25.8 - 27.9)	18.2 (15.3 - 36)	13.3 (12.6 - 26.5)	19.6 (-)	0.493
LA diametre z score, mean (SD)	+25.86 (7.3)	+28.84 (16.6)	+4.04 (1.32)	-	0.308
MLVWT (mm), median (25th-75th centile)	10.5 (8.5 - 14.5)	14 (10 - 18)	7 (6 - 10)	7 (6 - 8)	**0.002**
MLVWT z score, mean (SD)	+12.23 (6.9)	+16.65 (3.9)	+6.57 (0.4)	-	0.43
LVOT gradient (mmHg), median (25-75th centile)	36 (17 - 60)	43 (16 - 58)	55 (7.5 - 100)	6.5 (4.5 - 26.5)	0.232
LVOTO, n (%)	14 (63.6)	8 (61.5)	2 (50)	1 (12.5)	0.338
Mid cavity obstruction, n (%)	15 (68.2)	8 (80)	2 (33.3)	-	**0.003**
SAM, n (%)	17 (65.4)	9 (64.3)	3 (42.9)	1 (12.5)	0.073
RVH, n (%)	14 (60.9)	7 (53.9)	6 (75)	3 (37.5)	0.477
RVOT gradient (mmHg), median (25-75th centile)	18.5 (3.5 - 57.5)	21 (4 - 70.5)	16.5 (10 - 57)	2 (2 - 2.5)	0.401
RVOTO, n (%)	6 (50)	4 (57.1)	3 (50)	-	0.55
EF (%), median (25-75th centile)	79 (77.5 - 85.5)	86 (77.5 - 92.9)	79.5 (70.5 - 87)	80 (76.5 - 83.5)	0.703
Systolic dysfunction, n (%)	-	-	-	-	0.897
E/E' average, median (25-75th centile)	10 (7.2 - 12.9)	10.8 (7.3 - 28.3)	15.07 (-)	10.2 (9.6 - 11.6)	0.675
Diastolic dysfunction, n (%)	2 (18.2)	2 (66.7)	1 (100)	-	0.197

LVEDD: left ventricular end diastolic diameter, MLVWT: maximal wall thickness, LAd: left atrial diameter, LVOT: left ventricular outflow tract, LVOTO: LVOT obstruction, SAM: systolic anterior motion of the mitral valve, RVH: right ventricular hypertrophy, RVOT: right ventricular outflow tract, RVOTO: RVOT obstruction, EF: ejection fraction

Table S9: Electrocardiographic data at baseline assessment

		Total	%
Sinus rhythm		91	97.8
Left axis deviation		20	25.3
Right axis deviation		27	34.2
	Superior axis	21	44.7
PR interval prolongation		5	6.4
Right atrial enlargement		17	19.8
Left atrial enlargement		18	20.9
QTc prolongation		5	6.4
Voltage criteria for LVH		60	69.8
Conduction abnormalities	Intraventricular conduction delay	43	48.9
	RBBB	2	2.3
	LBBB	4	4.6
Pathological Q waves	Inferior leads	19	21.4
	Lateral leads	10	11.2
	Anterior leads	1	1.1
	>1 location	4	4.5
T wave inversion	Inferior leads	4	4.8
	Lateral leads	13	15.5
	Anterior leads	4	4.8
	>1 location	9	10.7
ST depression (<1mm)	Inferior leads	2	2.4
	Lateral leads	2	2.4
	Anterior leads	3	3.6
	>1 location	4	4.8
ST elevation (>2mm)	Absent	86	92.9
	Present	7	7.5

LVH: left ventricular hypertrophy, RBBB: right bundle branch block, LBBB: left BBB

Table S10: Outcomes by most prevalent genes

	PTPN11	RAF1	RIT1	HRAS	p value
Death, n (%)	3 (10.3)	1 (5.6)	-	2 (25)	0.44
Age at death (months), median (25th-75th centile)	3.3 (1.7 - 24)	5.26 (-)	-	12.4 (11.9 - 12.8)	0.651
Myectomy, n (%)	3 (10.3)	3(16.7)	1 (12.5)	-	0.37
ICD implantation, n (%)	2 (6.9)	1 (5.6)	1 (12.5)	-	0.889
CCF admission, n (%)	6 (20.7)	-	2 (25)	-	0.17
Heart transplant, n (%)	2 (6.9)	-	-	-	0.733
NSVT, n (%)	1 (3.3)	1 (5.6)	-	1 (12.5)	0.523
SCD or equivalent event, n (%)	2 (6.9)	1 (5.6)	1 (12.5)	1 (12.5)	0.316

n: number of patients, CCF: congestive cardiac failure, ICD: implantable cardiac defibrillator, NSVT: non-sustained ventricular tachycardia, SCD: sudden cardiac death

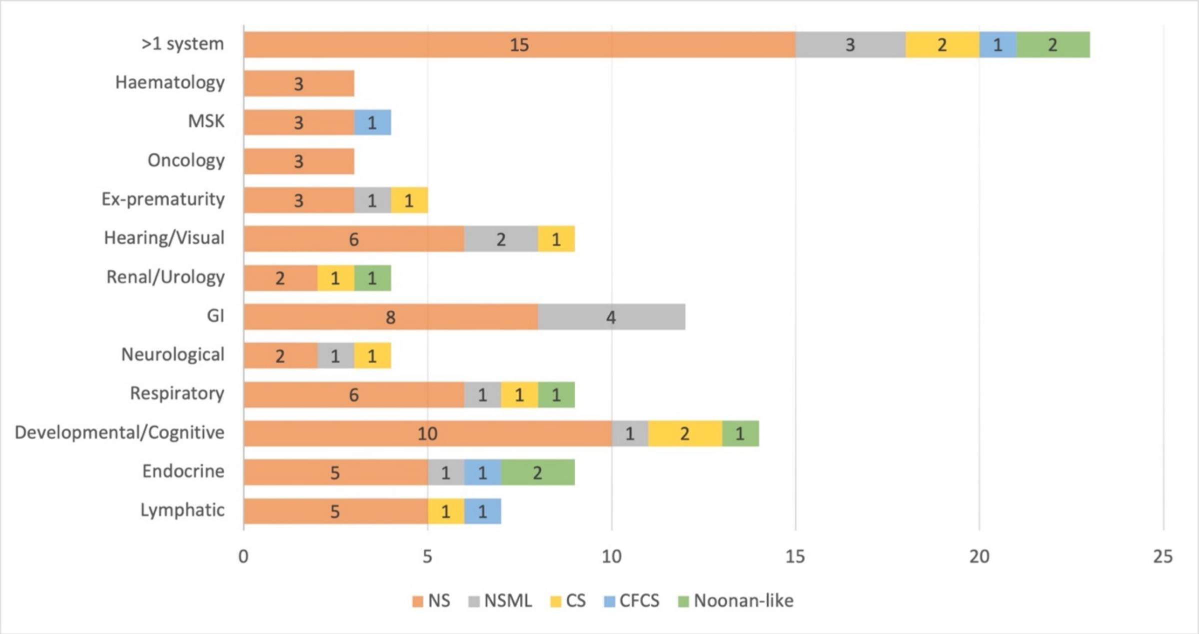

<u>Figure S1</u>: Extra-cardiac manifestations by RASopathy syndrome
X axis represents the absolute number of patients in each category by RASopathy syndrome and Y axis represents the system involved

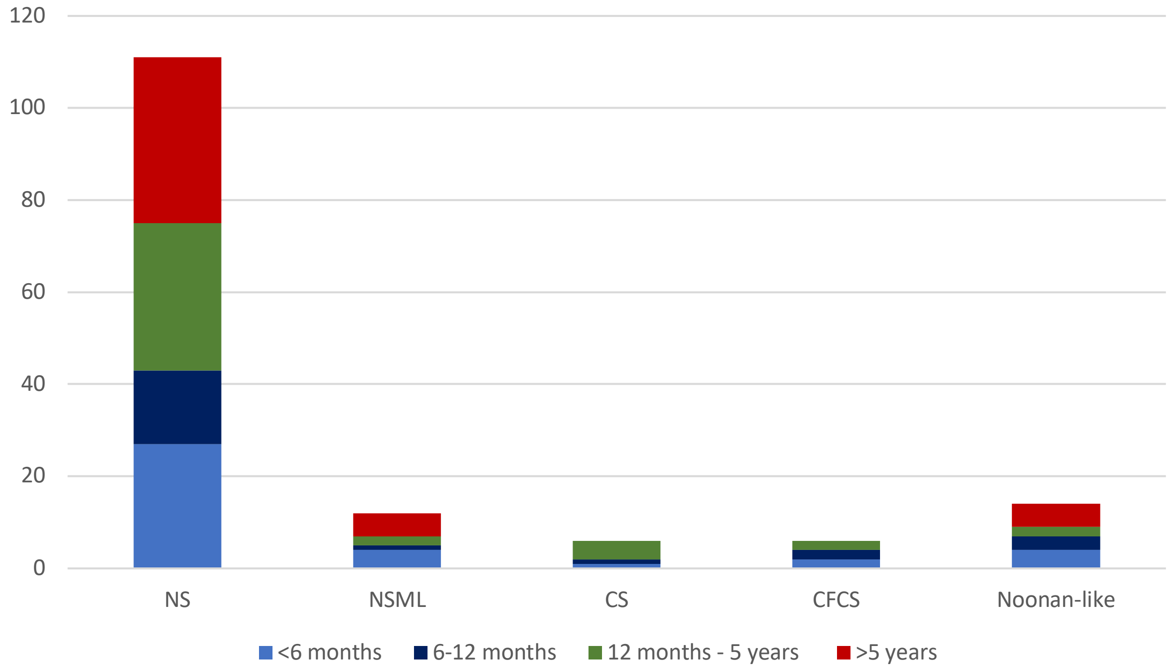

<u>Figure S2</u>: Age category by RASopathy Syndrome
X axis represents the different RASopathy syndromes and Y axis represents the absolute number in each age category

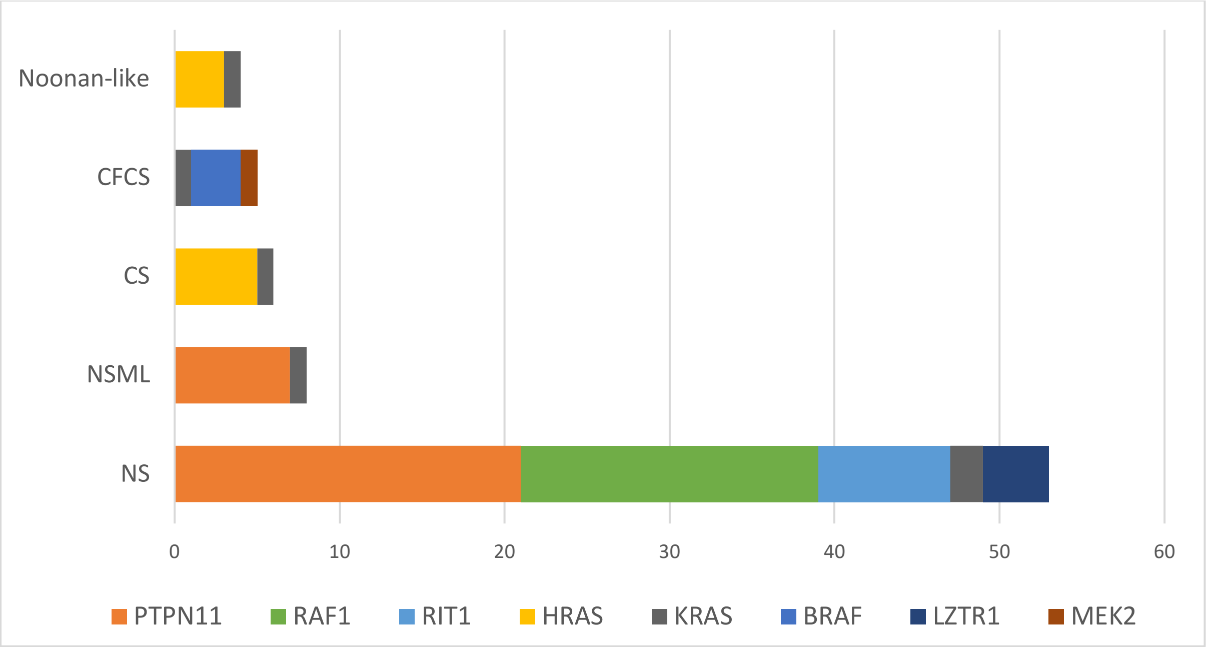

Figure S3: Gene mutation by RASopathy Syndrome
X axis represents the absolute number of each RASopathy syndrome by gene variant and Y axis represents each RASopathy syndrome

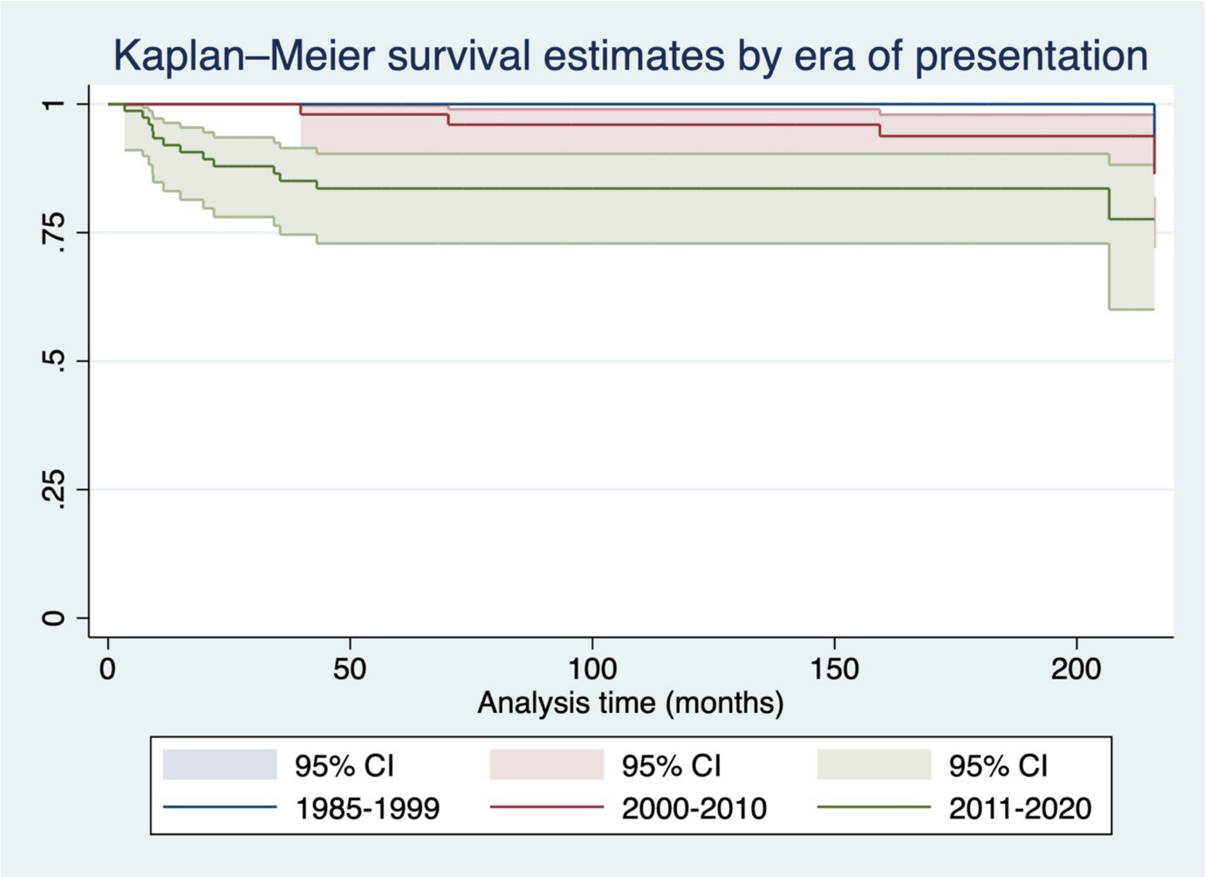

Figure S4: Kaplan-Meier survival estimates by era of presentation
(x) axis represents frequencies and (y) axis analysis time in months, 95% confidence intervals are shown for the corresponding curves in shading, (p = 0.453)
